# Recurrent Elsberg Syndrome Following Primary Herpes Simplex Virus Type 2 (HSV-2) Infection With Normal Spinal Imaging: A Case Report

**DOI:** 10.7759/cureus.112370

**Published:** 2026-07-09

**Authors:** Denise Nadora, Dawnica Nadora, Gavin Chima, Sharndeep Bains

**Affiliations:** 1 Neurology, California Northstate University College of Medicine, Elk Grove, USA; 2 Dermatology, California Northstate University College of Medicine, Elk Grove, USA; 3 Family Medicine, Jesuit High School, Arden-Arcade, USA; 4 Family Medicine, Rocklin Family Practice and Sports Medicine, Rocklin, USA

**Keywords:** cauda equina syndrome mimic, elsberg syndrome, herpes simplex virus type 2, lumbosacral radiculitis, neurogenic bladder, radiculomyelitis

## Abstract

Elsberg syndrome is a rare infectious neurologic condition characterized by acute or subacute lumbosacral radiculitis, with or without associated myelitis, most commonly linked to herpes simplex virus type 2 (HSV-2) or varicella zoster virus (VZV). Patients present with cauda equina-like symptoms, including urinary retention, saddle anesthesia, and neuropathic pain. Due to its variable diagnostic findings, the condition is often underrecognized.

We present the case of a 32-year-old woman with no prior neurologic history who developed progressive urinary retention, saddle anesthesia, genital sensory loss, and neuropathic pain following a primary genital HSV-2 infection. Initial magnetic resonance imaging of the thoracic and lumbar spine demonstrated no compressive lesion or abnormal enhancement. Cerebrospinal fluid analysis revealed a lymphocytic pleocytosis, though viral polymerase chain reaction testing was negative. Despite nondiagnostic imaging and laboratory findings, the clinical course was most consistent with HSV-associated lumbosacral radiculomyelitis (Elsberg syndrome). The patient was treated empirically with intravenous acyclovir and high-dose corticosteroids, resulting in gradual improvement in bladder function and neurologic symptoms, though residual sensory deficits persisted.

Approximately one year later, the patient presented with recurrent sacral pain, constipation, and concern for incomplete bladder emptying after the interruption of antiviral therapy. Repeat imaging again showed no evidence of cauda equina compression, and cerebrospinal fluid analysis demonstrated minimal inflammatory changes. Given her prior history, recurrent Elsberg syndrome was suspected. Symptoms improved rapidly following the re-initiation of antiviral therapy in combination with corticosteroids.

This case illustrates the variable and relapsing clinical course of Elsberg syndrome and highlights the importance of maintaining clinical suspicion in patients presenting with acute urinary retention and radicular symptoms following HSV infection, even in the absence of confirmatory imaging or cerebrospinal fluid findings. Early antiviral therapy and close longitudinal follow-up may be important in improving patient outcomes and preventing recurrence.

## Introduction

Elsberg syndrome is a rare infectious condition that presents as acute or subacute lumbosacral neuritis. While the exact etiology is not always clear, it is most commonly associated with herpes simplex virus type 2 (HSV-2) or varicella zoster virus (VZV). First described by Charles Elsberg in 1931, the syndrome typically manifests with lower back pain, bilateral lower extremity paresthesias, weakness, saddle anesthesia, and bladder or bowel dysfunction [1]. 

While it is estimated to account for 5-10% of cauda equina presentations, the true incidence is likely underreported due to limited awareness and inconsistent diagnostic testing [2]. Magnetic resonance imaging (MRI) may show lumbosacral nerve root enhancement or spinal cord abnormalities, but findings can also be normal [2]. Similarly, cerebrospinal fluid (CSF) analysis may reveal lymphocytic pleocytosis, yet viral polymerase chain reaction (PCR) testing may be negative despite active disease [2]. Thus, appropriate clinical correlation is crucial in diagnosing Elsberg syndrome. Early recognition is important because prompt antiviral therapy with acyclovir may reduce permanent neurologic symptoms and improve patient outcomes.

We present the case of a 32-year-old woman who developed progressive urinary retention, saddle anesthesia, genital sensory loss, and neuropathic pain following a primary genital HSV-2 infection. Despite repeatedly nondiagnostic imaging studies and inconclusive laboratory testing, her presentation was most consistent with HSV-associated lumbosacral radiculomyelitis (Elsberg syndrome). Her subsequent recurrence after the interruption of antiviral therapy further highlights the variable and potentially relapsing nature of this uncommon condition.

## Case presentation

A 32-year-old woman with no prior neurologic history presented with a five-day history of worsening dysuria, urethral burning, and external vulvar irritation. At her initial urgent care visit in February 2024, she reported a recent sexual encounter during which a condom was removed without her consent, raising concern for a sexually transmitted infection (STI). Laboratory testing returned positive for bacterial vaginosis and candidal vulvovaginitis, while STI screening, including HSV PCR, was negative. She was treated with metronidazole, fluconazole, doxycycline, and clotrimazole.

Over the next several days, her symptoms worsened. In March 2024, she returned with severe vaginal burning and the sudden appearance of painful ulcers on the right labia. Repeat testing demonstrated HSV-2 positivity from both ulcer and lesion swabs, confirming a genital herpes infection. She was advised that if the ulcers resolve, no further treatment is needed. However, if the ulcers remain, the patient was advised that a new antibiotic may be warranted.

Four days after the initial presentation, she began to experience persistent urinary urgency without the ability to void. Despite having an intense urge, she produced only small amounts of urine with significant straining. A diagnosis of HSV vulvovaginitis was made, and the patient was prescribed valacyclovir and referred to urology. A bladder ultrasound revealed 694 mL of retained urine, and she was instructed to begin intermittent self-catheterization while awaiting urgent urology evaluation.

Over the next several days, her urinary symptoms worsened and were soon accompanied by saddle numbness, genital sensory loss, and neuropathic pain. Given these concerning findings, she was admitted to the hospital 13 days after experiencing persistent urinary urgency without the ability to void, with a working diagnosis of possible cauda equina syndrome.

MRI of the thoracic and lumbar spine showed no compressive lesion, no abnormal enhancement, and a normal appearance of the cauda equina. A lumbar puncture revealed a lymphocytic pleocytosis, though cultures and viral PCR results were negative. Despite nondiagnostic testing, the clinical pattern strongly suggested HSV-associated lumbosacral radiculitis and myelitis, also known as Elsberg syndrome, triggered by her primary HSV-2 infection.

She was treated empirically with intravenous (IV) acyclovir and high-dose IV methylprednisolone, leading to gradual improvement in bladder function. By discharge nine days after hospital admission, she was voiding spontaneously with near-zero postvoid residuals, though she continued to experience reduced bladder sensation and saddle-region paresthesia. She remained on gabapentin for neuropathic pain and valacyclovir for suppression.

During the following months, her bladder function slowly improved. At the urology follow-up in April and October 2024, she demonstrated excellent bladder emptying (postvoid residual: 0-3 mL) but intermittently reported urinary urgency and diminished sensation. Neurology consultations later that year considered the etiology to be either direct HSV radiculitis or an immune-mediated postinfectious myelitis. She was advised that recurrence was possible but uncommon.

In June 2025, one year after her initial neurologic illness, she returned to the emergency department with acute sacroiliac pain, constipation, and concern about incomplete bladder emptying. She disclosed that she had missed approximately one week of her prophylactic valacyclovir regimen prior to symptom onset. She denied any genital lesions.

Evaluation revealed a mildly elevated erythrocyte sedimentation rate (ESR), a limited CSF sample showed only 5 white blood cells (WBCs) with a mixed differential, and there was no radiographic evidence of cauda equina compression. Given her history, these symptoms raised suspicion for recurrent Elsberg syndrome. A trial of IV steroids alone provided no significant improvement, but upon the initiation of IV acyclovir plus corticosteroids, she experienced rapid and notable relief. By discharge 28 days later, her pain had decreased to 2-3 out of 10, and she had returned to normal ambulation and lumbar range of motion. A summary of cerebrospinal fluid findings is presented in Table 1.

**Table 1:** CSF findings during the initial presentation and recurrence CSF: cerebrospinal fluid; WBC: white blood cell; HSV: herpes simplex virus; PCR: polymerase chain reaction

CSF parameter	Initial (March 2024)	Recurrence (June 2025)	Interpretation
WBC count	Lymphocytic pleocytosis	5 WBCs with mixed differential	Supports inflammatory process; improvement over time
Differential	Lymphocyte-predominant	Mixed cells	Consistent with resolving viral or postinfectious inflammation
HSV PCR	Negative	Negative	Does not exclude HSV-associated radiculomyelitis
Other viral PCRs	Negative	Negative	Viral involvement may persist despite negative testing
CSF cultures	Negative	Negative	No evidence of bacterial infection

She was transitioned to pregabalin for neuropathic discomfort and continued daily valacyclovir suppression. At follow-up, she had no further urinary retention and remained stable under outpatient neurology and urology monitoring.

This case demonstrates the complex clinical evolution of Elsberg syndrome from the initial genital HSV-2 infection to acute neurogenic bladder, followed by long-term sensory disturbances and eventual recurrence. It highlights how patients may present with urinary retention and radicular symptoms long before imaging or CSF findings confirm the diagnosis, emphasizing the need for clinical vigilance and early antiviral therapy in suspected cases of HSV-related lumbosacral radiculitis.

## Discussion

Elsberg syndrome is a rare neurological disorder that causes lumbosacral radiculitis, an inflammation of the nerves of the lumbosacral area of the spine, with possible myelitis. It differs from other infectious and autoimmune causes of cauda equina-like syndromes because it is specifically linked to a primary HSV-2 or a VZV infection or reactivation of the virus in the dorsal root ganglia [3,4]. It is often misdiagnosed as cauda equina syndrome due to its rare occurrence, leading to a delay in treatment and potential neurological sequelae. 

The mechanisms underlying Elsberg syndrome are thought to arise from a combination of direct viral neuroinvasion, local and systemic immune-mediated inflammation, and injury to the lumbosacral nerve roots, spinal cord, and autonomic pathways. Radiculitis is due to the virus infecting and replicating within the lumbosacral nerve roots. This leads to local inflammation, axonal injury, and, ultimately, demyelination. This damage is visualized by nerve root swelling and enhancement on MRI [4]. In this patient's case, the MRI showed no evidence of nerve root enhancement or spinal cord signal abnormality, highlighting the variant nature of Elsberg syndrome. Additional findings of an inflammatory process are also supported by lymphocytic pleocytosis in the CSF [3]. 

Myelitis, which may or may not be a presenting symptom of Elsberg syndrome, typically occurs when the virus spreads to the spinal cord. This may cause focal or multifocal myelitis in the lower thoracic or conus regions [3]. A study by Savoldi et al. revealed that myelitis occurs in Elsberg syndrome far more frequently than initially believed. While earlier literature suggested Elsberg was mostly radiculitis, this study showed that the cord is often involved. It explained that when there is myelitis, the spinal cord lesions were non-expansile and located centrally or ventrally and, often, did not affect the distal conus [3]. 

Autonomic dysfunction, which manifests as urinary retention, constipation, and sexual dysfunction, is due to the involvement of the autonomic fibers within the affected nerve roots and spinal cord segments. The sacral spinal cord segments S2 to S4 contain the parasympathetic preganglionic fibers responsible for activating detrusor muscle contraction and initiating normal bladder emptying [5]. Injury to these fibers disrupts normal autonomic control, as seen in the presenting case. Altogether, symptoms of radiculitis, myelitis, and autonomic dysfunction overlap as symptoms of urinary retention, constipation, saddle anesthesia, lower limb paresthesia, and pain [4].

Elsberg syndrome is diagnosed primarily through a combination of clinical history, presenting symptoms, and relevant imaging studies. In 2017, a criteria system was also suggested by Savoldi et al. [3]. According to the criteria, the presenting patient had clinical signs and symptoms of cauda equina involvement (urinary retention) with documented herpes virus infection, as defined by the "Laboratory-supported definite" category [3]. These findings align with the proposed diagnostic framework and further strengthen the diagnosis in this patient.

First-line treatment and management of viral etiologies, including Elsberg syndrome, is typically antiviral with IV acyclovir or valacyclovir. Early initiation, ideally within days of symptom onset, is associated with improved prognosis. Despite this, a study by Suarez-Calvet et al. reported outcomes ranging from full recovery to death despite antiviral therapy [6]. Additionally, adjunctive immunomodulatory therapy, including corticosteroids and immunoglobulin therapy, has also been associated with relieving urinary symptoms [7]. Supportive management, such as bladder catheterization, pain control, and rehabilitation for motor deficits, may also be included to help improve quality of life [7]. Lastly, management of relapses is prompt re-initiation of antiviral therapy, as seen in the presenting case. Therefore, with the possibility of relapses and persistent deficits, long-term close monitoring and follow-up are crucial to improve patient outcomes.

In this case report, the patient presented with classical symptoms seen in Elsberg syndrome, including acute urinary retention, persistent urinary urgency with impaired bladder sensation, saddle anesthesia, genital sensory loss, and a confirmed HSV-2 genital infection. Additionally, improvement with antiviral medication strongly suggests the diagnosis of Elsberg syndrome. However, there are other features that are missing in this patient, including a lack of clear MRI abnormalities and no documented bowel incontinence or lower extremity weaknesses. While these features are not necessary to diagnose Elsberg, this does highlight how variable Elsberg can present and how heavily diagnosis relies on clinical judgement.

## Conclusions

This case demonstrates the diverse clinical manifestations of Elsberg syndrome. Early antiviral therapy was key to this patient's improvement and recovery. Her recurrent symptoms further emphasize the potential for relapse and the need for long-term follow-up. Increasing clinician awareness may help reduce diagnostic delays and improve outcomes in similar presentations.
